# Evidence of Insecticide Resistance to Pyrethroids and Bendiocarb in* Anopheles funestus* from Tsararano, Marovoay District, Madagascar

**DOI:** 10.1155/2018/5806179

**Published:** 2018-10-08

**Authors:** Tsiriniaina Rakotondranaivo, Solohery Fanomezana Randriamanarivo, Mihajarilala Rakotoniaina Tanjona, Inès Vigan-Womas, Milijaona Randrianarivelojosia, Mamadou Ousmane Ndiath

**Affiliations:** ^1^Institut Pasteur de Madagascar, G4 Malaria Group, Ambatofotsikely, Antananarivo 101, Madagascar; ^2^Ecole Doctorale Génie du Vivant et Modélisation, Université de Mahajanga, Mahajanga 401, Madagascar; ^3^Institut Pasteur de Madagascar, Unité d'Immunologie des Maladies Infectieuses, Ambatofotsikely, Antananarivo 101, Madagascar; ^4^Institut Pasteur de Madagascar, Unité de Recherche sur le Paludisme, Ambatofotsikely, Antananarivo 101, Madagascar; ^5^Faculté des Sciences, Université de Toliara, Toliara 601, Madagascar

## Abstract

**Introduction:**

In Madagascar, malaria control relies on the countrywide use of long lasting insecticide treated bed nets (LLINs) and on indoor residual spraying (IRS) in the central highland area as well as a small area on the eastern coast. We tested insecticide resistance mechanisms of* Anopheles funestus* from Tsararano, a malaria endemic village in the coastal health district of Marovoay.

**Methods:**

Insecticide susceptibility bioassays were done in July 2017 on first-generation* Anopheles funestus *(F1) to assess (i) the susceptibility to permethrin (0.05%), deltamethrin (0.05%), DDT (4%), malathion (5%), fenitrothion (1%), and bendiocarb (0.1%); (ii) the effect of preexposure to the piperonyl butoxide (PBO) synergist; and (iii) the enzymatic activities of cytochrome P450, esterases, and glutathione S-transferases (GST).

**Results:**

Our results demonstrated that* An*.* funestus *was phenotypically resistant to pyrethroids and bendiocarb, with a mortality rate (MR) of 33.6% (95%CI: 24.5-43.7%) and 86% (95%CI: 77.6-92.1%), respectively. In contrast,* An*.* funestus* were 100% susceptible to DDT and organophosphates (malathion and fenitrothion). Preexposure of* An*.* funestus* to PBO synergist significantly restored the susceptibility to bendiocarb (MR=100%) and increased the MR in the pyrethroid group, from 96% (95%CI: 90.0-98.9%) to 100% for deltamethrin and permethrin, respectively (*χ*^2^ = 43,* df* = 3,* P< 0.0001*). Enzymatic activities of cytochrome P450 and *α*-esterases were significantly elevated among* An*.* funestus* compared with the IPM* reference *strain (Mann-Whitney* U*= 30*, P<0.0001*;* U *=* 145.5, P <0.0001*, respectively). No significant differences of *β*-esterases activities compared to the IPM reference strain were observed (Mann-Whitney* U = 392.5, P = 0.08*).

**Conclusion:**

In Tsararano, despite the absence of an IRS programme, there is evidence of high levels of insecticide resistance to pyrethroids and bendiocarb in* An*.

## 1. Background

Malaria remains a major public health burden on the island of Madagascar. Today, despite the fight against malaria which is mainly based on the use of rapid diagnostic test (RDT), the administration of artemisinin-based combination therapies (ACTs) as first-line treatment, and the implementation of vector control measures, malaria remains the third leading cause of morbidity and mortality in Madagascar, behind respiratory infections and diarrhea [1]. In 2015, malaria morbidity was 10.1% for all ages and 18.6% for children under 5 years of age. The malaria-related mortality ranged from 12.3% to 25.7% and incidence from 3.1% to 6.7% among those under 5 years of age [2, 3]. Thus, malaria still hampers the prosperity and economic development of the country as the economic cost of malaria is estimated at more than 50 million USD per year [4]. Consequently, in Madagascar, these malaria control measures must be increased to achieve the global malaria action plan (GMAP) [5].

Madagascar deployed traditional vector control methods as early as 1949 [6, 7]. The global malaria eradication campaign in the 1950s and 1960, which was based on the large-scale use of dichlorodiphenyltrichloroethane (DDT), was successful in Madagascar and there was a significant drop in national malaria transmission from 1959 until the early 1970s [8]. Unfortunately, these efforts were not sustained and recurring malaria outbreaks were observed in the central highlands in the 1980s [9, 10]. The upsurge of the disease was due to several factors such as the abandonment of indoor residual spraying (IRS), insufficient funding for malaria control, the erosion of the public health system, and the poor knowledge of the malaria vector biology (including that of* Anopheles funestus,* the main vector involved in the malaria epidemics in the central highlands) as well as the* Anopheles *resistance to insecticides [11–13].

The diversity of Madagascar's ecosystems with 5 epidemiological zones, several vectors, 2 major parasites (*P. falciparum* and* P. vivax*), and the changes they experience as a result of human activities (e.g. the use of insecticides for agriculture and vector control), among others, renders the malaria vector system extremely complex and constantly evolving. Today, it is widely accepted that vector control strategies will only be effective in the long term if there is a thorough knowledge of vector biology and, particularly, the status of insecticide resistance [14, 15]. The failure to take into account insecticide susceptibility of the potential vectors involved in malaria transmission has contributed to failures in malaria control throughout Africa [5].

The most effective way to prevent malaria transmission is to avoid human-vector contact, hence the importance of vector control [5]. However, in many countries where vector control strategies, including IRS and long lasting insecticide treated bed nets (LLINs), have been implemented on a large scale, profound vector changes have been observed. These range from behavioral changes to insecticide resistance, which greatly undermine the success of current vector control [16, 17]. This situation is all the more compelling when considering that* Anopheles *exhibits a strong resistance to pyrethroids [5], the only insecticide recommended and approved by the WHO for bed net impregnation [18]. In several countries, resistance to pyrethroids has been reported in major malaria vectors including* An*.* gambiae *s.l. and* An*.* funestus *[19, 20]. This has become an almost universal problem and may seriously impair the progress noticed in fighting malaria [5].

In Madagascar, IRS using DDT has been implemented since the 1950s whereas LLINs were widely distributed in the last decade [9, 21]. Malaria control still relies on the use of LLINs throughout the country and on IRS in the central highland area and in a small area on the eastern coast [22].

In malaria endemic regions, mosquito resistance can increase rapidly following the implementation of vector control [17]. Therefore, it is crucial to monitor the resistance to insecticides, particularly in countries where the policy of universal bed net coverage is applied. Recent studies demonstrated the occurrence of the resistance to pyrethroids and DDT in* An. arabiensis* population in several districts of Madagascar [23]. However, little information is available on the status and insecticide resistance mechanisms in* An*.* funestus*, one of the main malaria vectors [24]. In this paper, we report the insecticide susceptibility in wild* An*.* funestus* population from Tsararano and the involved resistance mechanisms.

## 2. Methods

### 2.1. Study Site

**Figure 1:**
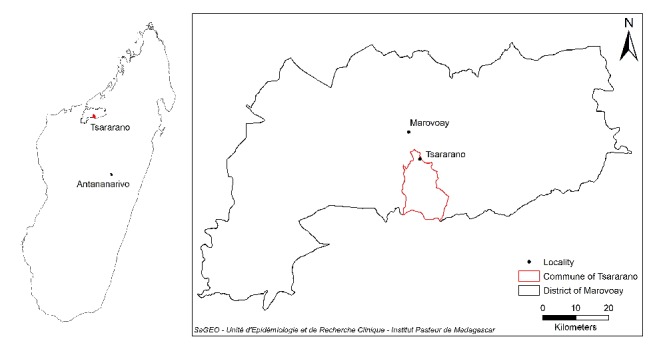
Map of Marovoay district (Madagascar) showing study Tsararano area.

**Figure 2:**
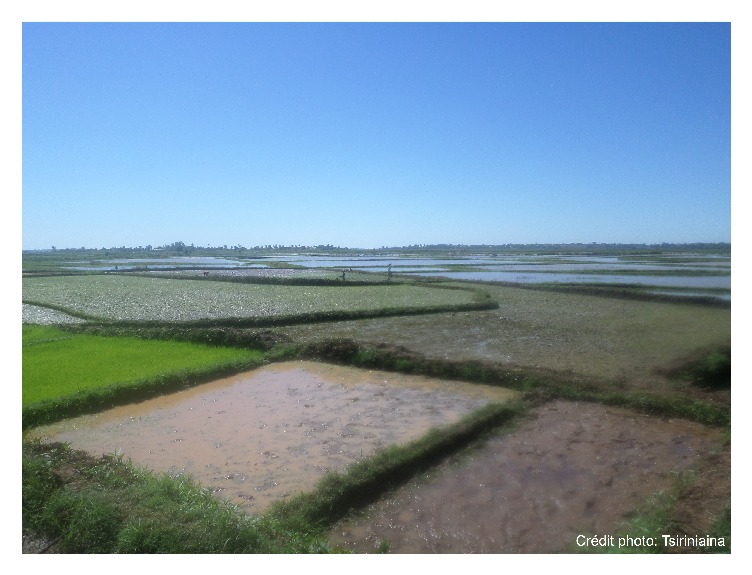
Photograph showing the vast rice fields at Tsararano Marovoay constituting potential larval breeding site for* Anopheles funestus*.

This study was carried out in the village of Tsararano (S 16°10′42.4′′, E 046°40′13.6′′), located in the district of Marovoay, in the Boeny region, approximately 540 km northwest of Antananarivo, the capital city of Madagascar (Figure 1). The village has about 1,200 inhabitants. It has a tropical climate and rainfall occurs from October to April. The average annual temperature is 29°C and the average rainfall is 1,500 mm. Tsararano is situated on the marshy bank of a permanent stream river, the Betsiboka. In this second largest rice granary region of Madagascar, agriculture is the main activity (Figure 2).* Anopheles* larval sites are present all year round and malaria is endemic. No IRS has been implemented in the district since the 1950s; however, LLINs were distributed for the fifth round in December 2015.

### 2.2. Mosquito Collection

Blood fed female mosquitoes were collected in the morning between 6 and 10 am from ten (10) households during the dry season in July 2017, using a mouth aspirator and transferred at the insectary of CSB2 Andriba platform. After morphological identification [25], one hundred and eighty-six (186) blood fed* An*.* funestus* were individually transferred into a tube (Eppendorf®) for forced oviposition according to the method described by Nepomichene* et al.* [26]. In brief, female mosquitoes were maintained at 28°C in 75-80% relative humidity with free access to 10% sucrose until they became fully gravid (about four days) and individually placed inside a tube with a filter paper placed at the bottom. Mosquitoes were introduced inside the tube one by one from within the cage. Oviposited eggs from each female were counted and pooled and were subsequently placed into a rearing pan containing well-water. Pooled larvae of different stages L1, L2, L3, and L4 were fed daily with Tetramin™ baby fish food. Pupae were collected and placed in 2-L plastic buckets, which were covered with mosquito gauze with a cotton sleeve for introducing 10% glucose on filter paper and allowed to emerge locally. Unfed 2-5-day females on first-generation* An. funestus* (F1) were used for insecticide susceptibility bioassay.

### 2.3. Susceptibility Assays

Insecticide susceptibility bioassays, following WHO protocol [27], were done on F1* An*.* funestus *generation at the insectary of Andriba platform. Six insecticides of technical grade quality were tested: two pyrethroids (permethrin 0.75%, deltamethrin 0.05 %), two organophosphates (malathion 5%, fenitrothion 1%), one organochlorine (DDT 4%), and one carbamate (bendiocarb 0.1%). Impregnated papers were obtained from the WHO reference center (Vector Control Research Unit, University Sains Malaysia, Penang, Malaysia). Tests were performed with batches of 25 unfed females aged 2-5 days, fourfold with each insecticide. Mosquitoes were exposed for 1 hour at 28°C and 80% relative humidity. After exposure, mosquitoes were kept in observation tubes and supplied with a 10% sugar solution. The number of knockdown (KD) mosquitoes was recorded at 10, 15, 20, 30, 40, 50, and 60 min. Mortality rate (MR) was recorded after 24 hours. Two batches of 25 females exposed to untreated papers were used as negative control. All batches of insecticide paper used were pretested on the strain of* An. arabiensis *from the Institut Pasteur de Madagascar known to be susceptible to all insecticides [28].

### 2.4. PBO Synergist Assays

In the event of confirmed insecticides resistance, additional tests were conducted by using the synergist piperonyl butoxide (PBO), an inhibitor of oxidases. Three combinations were used: PBO + deltamethrin, PBO + permethrin, and PBO + bendiocarb. For each combination, two exposed batches and one negative control batch exposed only to PBO were used. Each batch was constituted by 25 unfed female mosquitoes aged 2-5 days old. Mosquitoes were first exposed for one hour to 4% PBO and second to impregnated papers. KD mosquitoes and mortality rate were recorded as described above.

### 2.5. Biochemical Enzyme Assays

A subset of F1* An. funestus *generation (nonexposed to insecticide) stored at -80°C until use was used for biochemical enzyme assays. Enzymatic activities of cytochrome P450, esterases (*α* and *β*), and glutathione S- transferases (GST) were measured according the protocol described by Brogdon* et al. *[29] and by Hemingway* et al. *[30], and slightly modified by Sangba* et al. *[31]. Absorbance was measured using a spectrophotometer type “Multiskan GO and SkanIt Software/Serial number 1510-05171” (https://www.thermofisher.com/us/en/home/life-science/lab-plasticware-supplies/lab-plasticware-supplies-learning-center/lab-plasticware-supplies-resource-library/skanIt-software-protocols-multiskan-go.html). In the absence of* An*.* funestus* susceptible strain, the* An*.* arabiensis *strain from the Institut Pasteur de Madagascar known to be susceptible to all insecticides was used as control [28]. All experiments were conducted at the laboratory of the Institut Pasteur de Madagascar.

### 2.6. Species Molecular Identification

DNA was extracted from all F0 individual mosquitoes by DNAzol (Invitrogen, CA, USA) according to the manufacturer's recommendations.* Anopheles *mosquitoes were identified by the PCR using the method described by Santolamazza* et al. *for* An*.* gambiae*, and the technique described by Wilkins* et al. *for* An*.* funestus *[32]. The PCR for* An*.* gambiae *s.l. discrimination is based on the amplification of a ribosomal DNA fragment smaller amplified by PCR by using the following primers: IGS441 (F) [TGG TCT GGG GAC CAC GTC GAC ACA GG], IGS783 (R) [CGT TTC TCA CAT CAA GAC AAT CAA GTC], while the PCR for* An*.* funestus* discrimination is based on species-specific single nucleotide polymorphism (SNPs) in the second internal transcribed spacer region (ITS2) by using seven primers: UV (F,) [CCG ATG CAC ACA TTC TTG AGT GCC TA], FUN (R) [CTC GGG CAT CGA TGG GTT AAT CAT G], VAN (R) [AAC TCT GTC GAC TTG GTA GCC GAA C], RIV (R) [AAT CAG GGT CGA ACG GCT TGC CG], PAR (R) [GCC CTG CGG TCC CAA GCT AGA TT], RIVLIKE (R) [CTC CCG TGG AGT GGG GGA TC], LEES (R) [GAC GGC ATC ATG GCG AGC AGC]. All primers were provided by the Center for Diseases Control (CDC) and the experiments were done at the laboratory of Institut Pasteur de Madagascar.

### 2.7. Data Analysis

For each insecticide, the percentage of KD and MR was determined. Since mortality in negative controls was always under 5%, no adjustment was performed for treated batches. The WHO 2017 criteria were used to evaluate the status of insecticide resistance (i.e., 98-100% mortality indicates susceptibility and <98% mortality indicates that further investigation is required to confirm resistance) [27]. Fifty and ninety-five percent KD times (KDT_50_ and KDT_95_, respectively) were computed with probit regression models. Mortality rates expressed in (%) and 95% confidence interval were compared using Fisher exact test. The enzymatic activities of wild* An*.* funestus* were compared with that of the* An*.* arabiensis *strain from the Institut Pasteur de Madagascar known to be susceptible to all insecticides by Mann-Whitney test. Statistical analyses were performed using GraphPad® Prism software v5.0 (www.graphpad.com). A* P* value of 0.05 or less was considered as significant.

## 3. Results

### 3.1. Wild Anopheles Collections

In July 2017, a total of 311 malaria vectors were collected from 10 households in Tsararano. Among those, 254 (81.7%) were* An*.* funestus* and 57 (18.3%)* An*.* gambiae* s.l. (Table 1). Most of these mosquitoes were blood-fed. From the 186 oviposition tubes set up with individual gravid* An*.* funestus* females, 175 produced egg batches. The total number of eggs collected was 12,011, averaging 64.57 eggs per female.

### 3.2. Molecular Identifications and Infection Rates

All F0* An*.* funestus* group mosquitoes (n=254) tested by PCR showed that they all belonged to* An*.* funestus *s.s. Of the 57* An*.* gambiae *s.l. tested, 96.5% (n=55) were identified as* An*.* arabiensis* and 3.5 % (n=2) as* An*.* gambiae *(formerly S molecular form) (Table 1).

The* Plasmodium falciparum* circumsporozoite protein (CSP) rate was determined by ELISA-CSP. Of the 311* Anopheles* specimens tested by ELISA-CSP, 4 were positive for the CSP antigen, resulting in a mean CSP rate of 1.3% (95%CI: 0.35–3.2%) with 1.2% and 1.8% for* An. funestus* and* An. gambiae *s.l., respectively (*χ*^2^ = 0.12,* df* = 1,* P = 0.7*).

### 3.3. Susceptibility of Anopheles funestus to Insecticides

**Figure 3:**
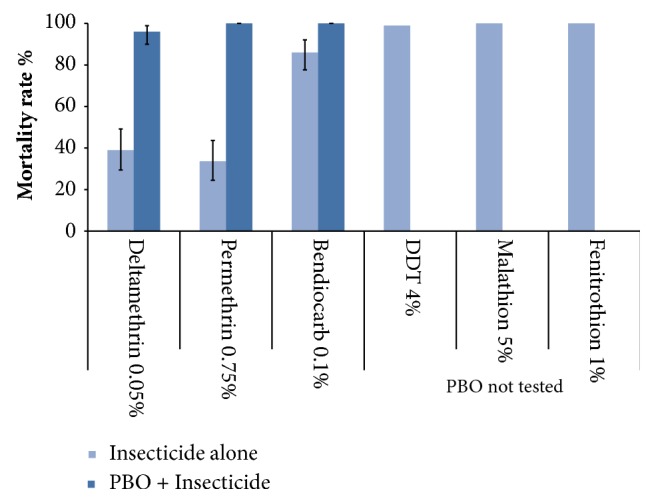
Insecticide bioassay mortality rate in wild* Anopheles funestus *first generation (F1) from Tsararano, Marovoay (Madagascar), 24 hours after exposure to permethrin (0.75%), deltamethrin (0.05%), DDT (4%), malathion (5%), fenitrothion (1%), and bendiocarb (0.1%) and effect of piperonyl butoxide (PBO). The data represent medians.

Table 2 summarizes the MR of* An*.* funestus* population induced by all insecticides. Following 24 h observation, MRs indicated high resistance to pyrethroids and bendiocarb (Figure 3 and Table 2). The mean MR induced by permethrin and deltamethrin in the wild* An. funestus* population was 33.6% (95%CI: 24.5–43.7%) and 39% (95%CI: 29.4–49.2%), respectively. No significant difference of the MR was observed in the pyrethroid group (*χ*2 = 0.61,* df* = 1,* P*= 0.4). The mean MR induced by bendiocarb in the wild* An*.* funestus* population was 86% (95% CI: 77.1 – 92.1%).

*An. funestus *was 100% susceptible to DDT and organophosphate (malathion and fenitrothion) (Figure 3 and Table 2). Furthermore, 100% of mosquitoes were knockdown after 60 min exposure to malathion, fenitrothion, and DDT with a short KDT_50_ time <28.9 min (95%CI: 25.7-32.2) (Table 2). For pyrethroids the KDT_50_ was much longer (higher than 72 min) (Table 2). Meanwhile, a low KDT_50_ was observed after bendiocarb exposure despite the resistance (Table 2).

### 3.4. Effects of Synergist PBO on Anopheles funestus Resistant to Pyrethroids and Bendiocarb

For both pyrethroids and bendiocarb, preexposure to PBO synergist significantly increased the MR (Figure 3). The mean MR of deltamethrin increased from 39% (95%CI: 29.4–49.2%) before PBO exposure to 96% (95%CI: 90–98.9%) after PBO exposure. The MR induced by permethrin increased from 33.6% before PBO exposure to 100% after PBO exposure. A significant difference of MR was observed in pyrethroid group before and after PBO exposure (*χ*^2^ = 43,* df = 3, P< 0.0001*). Concurrently, a full susceptibility was observed to bendiocarb after PBO exposure (MR=100%) (Figure 3).

### 3.5. Biochemical Enzymatic Activities of Cytochrome P450, Esterases, and GST

**Figure 4:**
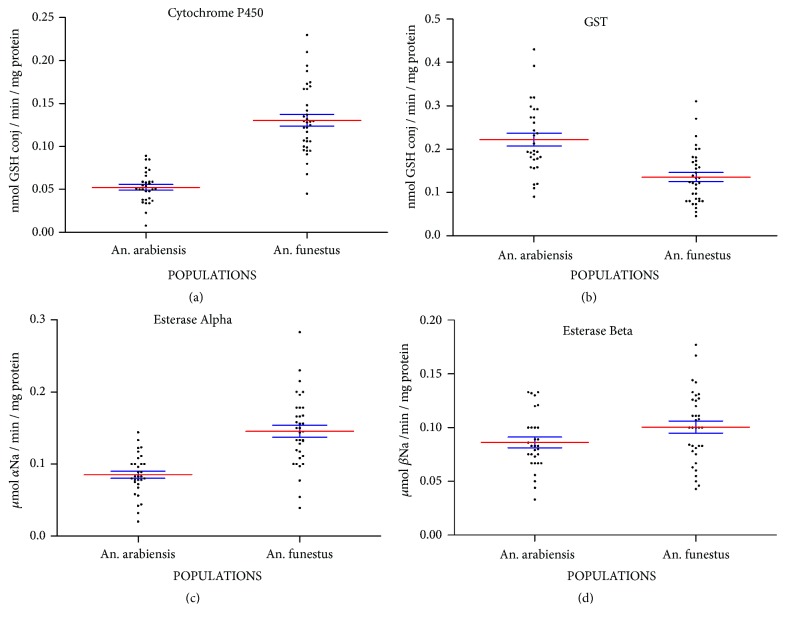
Detoxifying enzyme activities (enzymatic activity per mg of protein) of wild* Anopheles funestus *first generation (F1) from Tsararano, Madagascar, in comparison with* Anopheles arabiensis* Institut Pasteur of Madagascar susceptible strain. (a) Cytochrome P450 activities (MFO). (b) Glutathione S-transferases activities (GST). (c) Alpha esterase activities. (d) Beta esterase activities.* Red* lines represent means with 95% confidence intervals (blue lines).

Biochemical data of enzymatic activities of cytochrome P450, GST, and esterases (*α* and *β*) compared to the IPM susceptible strain are shown in Figure 4. High level enzymatic activities of cytochrome P450 compared to the susceptible IPM reference strain were observed (Mann-Whitney* U*= 30,* Z = -6.51, P<0.0001*) (Figure 4(a)). In contrast, the GST activity was significantly lower compared to the IPM reference strain (Mann-Whitney* U = 195.5, Z = -4.33, P <0.0001*) (Figure 4(b)) suggesting the no implication of GST in the insecticide resistance of* An*.* funestus* from Tsararano.

Meanwhile, a significant increased *α*-esterase activity was observed in the* An*.* funestus* population compared to the IPM susceptible strain (Mann-Whitney* U*=* 145.5, Z = -4.99*,* P <0.0001*) (Figure 4(c)). In contrast, no significant *β*-esterase activity was observed in the* An*.* funestus* populations from Tsararano compared to the IPM strain (Mann-Whitney* U = 392.5, Z = -1.87, P = 0.08*) (Figure 4(d)).

## 4. Discussion

This study, conducted in Tsararano, reports the levels of insecticide susceptibility of* An*.* funestus*, a main malaria vector in Madagascar, to the different families of insecticides conventionally used in vector control in the country.

Our study showed that* An*.* funestus* is the only species of the funestus group present in the study area with a moderate index circumsporozoite rate, which was considerably smaller than that obtained by Marrama* et al.* [13], and Fontenille* et al.* [11] in other parts of Madagascar. However, in many sub-Saharan Africa regions, entomological inoculation rate (EIR) of* An*.* funestus* surpasses that of* An. gambiae*, indicating their major role in malaria transmission throughout the continent [17, 33].

The study provides substantial knowledge on the resistance status of the wild* An*.* funestus* population. The results of insecticide bioassay trials showed a high biological level of resistance to pyrethroid groups (deltamethrin and permethrin) and bendiocarb. Similar resistance has been observed in southern east Africa, particularly in Mozambique [34], but in Madagascar, little information on* An*.* funestus *is available, in contrast to* An*.* gambiae *[23, 24]. Furthermore, investigations on the biological insecticide susceptibility performed in sentinel sites of Madagascar in 2015 reported a resistance to pyrethroids and bendiocarb in 20/37 and 8/24 sites, respectively, for* An*.* gambiae*. Meanwhile, resistance to the pyrethroid group and bendiocarb was observed only in 1/5 and 1/3 sites, respectively, for* An*.* funestus*, including in the district of Marovoay [24]. However, previous studies conducted in the Central Island of Madagascar by Rakotondraibe* et al* [35] and Ratovonjato* et al* [36] showed a full susceptibility to pyrethroids and DDT in wild blood feed and semigravid* An. funestus *population (F0) in contrast to our study where F1 progeny was used according to the WHO protocol [27]. This susceptibility difference may be due by several factors including the intensive uses of pesticides in Tsararano rice fields before 1950s [37] and also the age and physiological status of mosquitoes (i.e., blood fed and/or semigravid). For insistence, it has been demonstrated that older mosquitoes are sometimes less resistant to insecticides, especially when resistance is conferred by the presence of a detoxifying enzyme, the activity of which may decline with age [38].

On the other hand, previous studies have shown that the occurrence of the* Anopheles *resistance to insecticides is partly explained by the use of pesticides in agricultural activities and vector control to combat malaria [39, 40]. Given that Marovoay is the second largest rice granary in Madagascar, Tsararano is surrounded by rice fields where insecticides are used for pest control for an extended period of time throughout the year. Up to today, no IRS has been performed in Tsararano as part of the national vector control strategy. We hypothesize that the increased resistance of* An. funestus *to pyrethroids and bendiocarb observed in Tsararano might be due to the use of pesticides in agriculture. Nevertheless, the causes of such widespread resistance remain unknown. However, we believe LLINs would have a limited effect in spreading resistance in this region, as described elsewhere [41].

The long-term efficacy of LLINs in reducing malaria morbidity has recently been questioned in western Africa [17]. In Madagascar, 2,761,550 insecticide-treated nets were distributed for free to the population in 2016 [42]. Nevertheless, a full susceptibility to organophosphates has been observed in* An*.* funestus *population; therefore, these insecticides should be used in priority if IRS should be performed in this area.

The presence or absence of* kdr* mutation in* An*.* funestus* population from Tsararano was not investigated. Several studies have shown the absence of this mutation in* An*.* funestus* populations [41, 43] unlike in* An*.* gambiae*, where the widespread distribution of* kdr-w* and* kdr-e* has been observed in many African regions [5, 44, 45]. In addition, to our knowledge, no* kdr* mutation was observed in* An*.* gambiae *populations from Madagascar [23].

Preexposure to PBO restored the full susceptibility to pyrethroids and bendiocarb. Similar observations have been made in Mozambique [34, 46]. Biochemical enzymatic activity data showed that cytochrome P450 activity was significantly higher in* An*.* funestus* population than the IPM strain indicating that the pyrethroid resistance in Tsararano is driven by the cytochrome P450 genes, as previously observed in Mozambique and in the Central African Republic [34, 46]. In addition, we hypothesize that overexpression of cytochrome P450s also confers a cross-resistance to carbamates. This situation is particularly likely if the pyrethroid resistance results from a change in a P450 regulatory region, rather than a mutation in a single P450 structural gene, which could produce cross-resistance to several insecticide classes including carbamates, as previously observed in Mozambique [34]. Interestingly, previous studies revealed that pyrethroid resistance in* An*.* funestus* population is mainly driven by key cytochrome P450s genes such as CYP6P9a, CYP6P9b, and CYP6M7 [47, 48]. However, the gene expression pattern of the detoxification linked to this cross-resistance is not well understood.

Furthermore, a significant overexpression activity of *α*-esterase was observed in* An. funestus *from Tsararano. However, no difference in *β*-esterase activity was observed. In contrast, GST activity was significantly lower. This confirms the susceptibility of* An*.* funestus* from Tsararano to DDT. In fact, the evidence of resistance to the two major classes of insecticides (pyrethroids and bendiocarb) in the* An*.* funestus* population from Tsararano may constitute a potential threat to the success of the malaria vector control programme in this region. In addition, given the wide distribution of* An*.* funestus* in Madagascar, further investigation in other districts through the exploration of resistance profile is needed. This will provide information to elaborate adequate vector resistance management.

## 5. Conclusions

Our study showed evidence of resistance to pyrethroids and bendiocarb in an* An. funestus* population. Enzymatic activities data indicated implication of cytochrome P450 genes in the resistance of* An*.* funestus* with a suspected cross-resistance between pyrethroids and carbamate. The coexistence of this cross-resistance in* An*.* funestus*, where confirmed, constitutes a serious concern for the future success of malaria control programme.

## Figures and Tables

**Table 1:** Anophelines collected in Tsararano by indoor aspirator and molecular identification by PCR and circumsporozoite protein rate (CSP). Mosquitoes were captured in July 2017 during the dry season in ten (10) households at early morning between 6 and 10 am.

**Morphological identification**			
Methods	***Anopheles funestus* s.l.**	***Anopheles gambiae* s.l.**	

Indoor aspirator mosquito catch	254	57	

**molecular identification**			
PCR species	***Anopheles funestus* s.s.**	***Anopheles arabiensis***	***Anopheles gambiae***

	254	55	2

***Plasmodium falciparum* circumsporozoite protein rate**			

Number of Positive ELISA	3	1	0
CSP rate (%)	1.2%	1.8%	0

**Table 2:** Mortality rate (MR) 24 hours after exposition obtained after WHO bioassay first-generation *An. funestus *population (F1) from Tsararano, Marovoay, and knockdown time 50 (KDT_50_) and knockdown time 95 (KDT_95_). Mortality rate represents mean with 95% confidence intervals (CI). na: not applicable.

	**Mortality rate (**%**)**		**Knockdown time 50**		**Knockdown time 95**	
	**MR (n)**	**95**%**CI**	**KDT**_**50**_	**95**%**CI**	**KDT**_**95**_	**95**%**CI**
**Permethrin 0.75**%	33.6 (101)	[24.5-43.7]	73.79	[70.4-77.1]	na	-

**Deltamethrin 0.05**%	39 (100)	[29.4-49.2]	72.5	[68.1-76.9]	na	-
**Bendiocarb 0.1**%	86(100)	[77.6-92.1]	38.1	[36.4-39.7]	na	-
**DDT 4**%	99 (101)	-	34.4	[32.4-36.4]	na	-
**Malathion 5**%	100 (99)	-	28.9	[25.6-32.2]	39.0	[36.9-42.0]
**Fenitrothion 1**%	100 (102)	-	26.1	[22.7-29.6]	48.7	[44.7-52.7]

## Data Availability

The mortality rate data used to support the findings of this study are included within the article and the enzymatic activities data used to support the findings of this study are available from the corresponding author upon request.
